# Viral and bacterial etiology of severe acute respiratory illness among children < 5 years of age without influenza in Niger

**DOI:** 10.1186/s12879-015-1251-y

**Published:** 2015-11-14

**Authors:** Adamou Lagare, Halima Boubacar Maïnassara, Bassira Issaka, Ali Sidiki, Stefano Tempia

**Affiliations:** Centre de Recherche Médicale et Sanitaire (CERMES), 634 Bld de la Nation, BP:10887, YN034- Niamey, Niger; Influenza Division, Centers for Disease Control and Prevention, Georgia, Atlanta USA; Influenza Program, Centers for Disease Control and Prevention, Pretoria, South Africa

**Keywords:** Niger, Severe acute respiratory illness, Virus, Bacteria, Etiology

## Abstract

**Background:**

Globally, pneumonia is the leading cause of morbidity and mortality in children, with the highest burden experienced in sub-Saharan Africa and Asia. However, there is a dearth of information on the etiology of severe acute respiratory illness (SARI) in Africa, including Niger.

**Methods:**

We implemented a retrospective study as part of national influenza sentinel surveillance in Niger. We randomly selected a sample of nasopharyngeal specimens collected from children <5 years of age hospitalized with SARI from January 2010 through December 2012 in Niger. The samples were selected from individuals that tested negative by real-time reverse transcription polymerase chain reaction (rRT-PCR) for influenza A and B virus. The samples were analyzed using the Fast Track Diagnostic Respiratory Pathogens 21plus Kit (BioMérieux, Luxemburg), which detects 23 respiratory pathogens including 18 viral and 5 bacterial agents.

**Results:**

Among the 160 samples tested, 138 (86 %) tested positive for at least one viral or bacterial pathogen; in 22 (16 %) sample, only one pathogen was detected. We detected at least one respiratory virus in 126 (78 %) samples and at least one bacterium in 102 (64 %) samples. Respiratory syncytial virus (56/160; 35 %), rhinovirus (47/160; 29 %) and parainfluenza virus (39/160; 24 %) were the most common viral pathogens detected. Among bacterial pathogens, *Streptococcus pneumoniae* (90/160; 56 %) and *Haemophilus influenzae* type b (20/160; 12 %) predominated.

**Conclusions:**

The high prevalence of certain viral and bacterial pathogens among children <5 years of age with SARI highlights the need for continued and expanded surveillance in Niger.

## Background

Acute respiratory infections (ARIs) are responsible for substantial morbidity and mortality globally, especially in children <5 years of age, and the highest burden is observed in developing nations [1]. In 2000, approximately 2.2 million ARI-associated deaths occurred among children <5 years of age of which 70 % were in Africa and Southeast Asia [2]. Both bacteria and viruses have been identified as the etiological agents of ARI. The viruses most frequently detected in children with ARIs include respiratory syncytial virus (RSV), influenza virus (INF) types A and B, adenovirus (AV), parainfluenza virus (PIV), human metapneumovirus (HMPV) and rhinovirus (RV) [3–5]; however, the clinical presentations of respiratory tract infections are similar, making it difficult to distinguish between etiologic agents without a laboratory diagnosis [6]. In addition, the interpretation of a viral detection is complicated by the fact that infections with multiple viruses are common in children with ARI and many viruses are frequently found in asymptomatic children [7]. *Streptococcus pneumoniae* and *Haemophilus influenzae* type b (Hib) are the most commonly isolated bacteria from ARI cases, while, other atypical pathogens such as *Mycoplasma pneumoniae* and *Chlamydophila pneumoniae* are less frequently reported [8–10]. *S. pneumoniae* and Hib are commonly identified in nasopharyngeal samples from asymptomatic children due to high rates of carriage; however, their identification from the nasopharynx is rarely indicative of invasive disease [11].

The viral and bacterial etiology of ARI has been well documented in countries from the Northern hemisphere [12–15]; however, few studies are available from Africa [16, 17]. In Niger, a sentinel surveillance system for influenza viruses was instituted in April 2009; however, no studies on the etiology of ARI have been conducted in the country. We aimed to document the prevalence of selected viral and bacterial infections among children <5 years of age hospitalized with severe acute respiratory illness (SARI) at selected hospitals in Niger from January 2010 through December 2012.

## Methods

### Study design and setting

Niger is a West African country with a Saharan climate characterized by four distinct seasons: the cold season from mid-December to mid-February, the dry and hot season from mid-February to May, the rainy season from June to September, and the hot season from October to mid-December [18, 19].

Since April 2009, influenza surveillance has been conducted among patients hospitalized with severe acute respiratory illness (SARI) at 8 sentinel sites located in 5 of the 8 regions of the country by the Centre de Recherche Médicale et Sanitaire (CERMES), the National Reference Laboratory for influenza. The influenza surveillance program in Niger has previously been described [19]. Briefly, all patients hospitalized at the participating sentinel sites that met the SARI case definition were eligible for enrollment. Verbal informed consent was obtained from all cases who were 18 years of age and older. Proxy informed consent was obtained from parents or legal guardians of minors. Patients who did not meet the case definition or did not provide verbal consent were not included.

A SARI case was defined as a hospitalized child <5 years of age with onset of cough or difficulty breathing within 7 days prior to admission, and at least one of the following danger signs: inability to drink or breastfeed, lethargy, vomiting everything, convulsions, nasal flaring, chest indrawing, stridor in a calm child or tachypnea [20].

A standardized questionnaire was administrated by clinical personnel, to record patients’ demographic characteristics and medical history. The questions included information on date of enrollment and symptom onset, sex, age and clinical symptoms. Nasopharyngeal (NP) swabs were collected from all enrolled cases and placed in cryovials containing virus transport medium (Copan kit, Italy). The specimens were kept refrigerated at 4 °C at the sentinel site and then transported twice weekly to CERMES for testing. Samples were aliquoted, screened for influenza A and B viruses by real-time reverse transcription polymerase chain reaction (rRT-PCR), and then stored at −80 °C.

### Sampling and laboratory procedures

We conducted a retrospective study on the etiology of influenza-negative SARI cases among children <5 years of age enrolled in influenza surveillance during January 2010 through December 2012 in Niger. We randomly selected (using random selection procedures available in Stata) a sample of 160 stored NP specimens from 742 SARI cases which had tested influenza A and B negative. This sample represented 21 % of the total cases aged <5 years enrolled during the study period. Only 5.5 % of SARI cases in the <5 year old age group were tested positive for influenza. Influenza-positive cases were mainly detected during the cold season [21].

Nucleic acid was extracted using the QIAamp mini kit (Qiagen, Germany) in accordance with the manufacturer’s protocol. An internal control (IC) was added to each extraction tube in order to assess the quality of the extraction at the end of the amplification. Extracted samples were screened by rRT-PCR with Fast Track Diagnostic (FTD) Respiratory pathogens 21plus kit (BioMérieux, Luxemburg) following the manufacturer’s procedure using six multiplex PCR for 18 viruses: influenza (INF) types A and B, parainfluenza virus (PIV) types 1–4, coronavirus (CV) NL63, 229E, OC43, and HKU1, metapneumovirus A-B (HMPV), respiratory syncytial virus A-B (RSV), parechovirus (PV), enterovirus (EV), adenovirus (AV), human bocavirus (HBV), rhinovirus (RV) and 5 bacterial pathogens: *Mycoplasma pneumoniae* (*M. pneumoniae*), *Chlamydophila pneumoniae* (*C. pneumoniae*), *Streptococcus pneumoniae* (*S. pneumoniae*), *Haemophilus influenzae* type b (Hib), and *Staphylococcus aureus* (*S. aureus*) [22]. Two different positive controls for viral and bacterial multiplex PCR reactions and a negative control tube are provided in the kit

### Statistical analysis

The *Χ*^*2*^ and the Fisher’s exact tests were used to assess the difference between categorical variables by comparing expected and observed frequencies across evaluated groups. In addition, we compared the characteristics of selected and non-selected children (including influenza-positive cases) using the *X*^*2*^ statistics. The statistical analysis was implemented using Stata version 13.1 (StataCorp®, Texas, USA).

### Ethics approval

In 2009, the National Ethics Committee of Niger approved the national influenza surveillance program (reference No06/2009/CCNE of April 2009). In 2012, prior to the investigation of other respiratory pathogens, the Ministry of Health provided an extended approval. Access to the study samples was granted by the Director of CERMES.

## Results

From January 2010 through December 2012 we enrolled 785 children <5 years of age hospitalized with SARI into the influenza surveillance program, of which 742 (94 %) tested negative for influenza virus. Of these, 160 (21 %) were randomly selected for our study. The age, sex and symptom duration distribution did not differ significantly among selected and non-selected children (including those that tested positive for influenza). However, among the selected group there was a significantly lower proportion of specimens collected during the cold season, when the majority of influenza-positive cases were detected and excluded from randomization (Table 1).

**Table 1:** Characteristics of children <5 years of age hospitalized with severe acute respiratory illness selected and non-selected for the study in Niger, 2010–2012

Characteristics	Children selected for the study	Children non-selected for the study^a^	*p*
	*n* (%)	*n* (%)	
	*N* = 160	*N* = 625	
Age (in years)			0.811
<1	90 (56.2)	362 (57.9)	
1–4	70 (43.8)	263 (42.1)	
Sex			0.121
Female	7 (45.6)	243 (38.9)	
Male	87 (54.4)	382 (61.1)	
Duration of symptoms (in days)			0.88
0–3	125 (78.1)	524 (83.8)	
4–7	35 (21.9)	101 (16.2)	
Season			<0.001
Hot	64 (40.0)	255 (40.8)	
Cold	6 (3.7)	97 (15.5)	
Rain	48 (30.0)	135 (21.6)	
Dry	42 (26.3)	138 (22.1)	

Abbreviations, *CI* confidence interval

^a^Including children that tested positive for influenza virus

Among the selected children, 56 % (90/160) were <1 year of age (median age among children age <5 years: 9 months), 46 % (73/160) were female and 78 % (125/160) had a duration of symptoms ≤3 days. Most patients presented with a recorded temperature >38 °C (58 %; 93/160), cough (96 %; 155/160), dyspnea (69 %; 111/160) and chest indrawing (76 %; 122/160). Few patients presented with tachypnea or had difficulty in breastfeeding (15 %; 25/160).

**Fig. 1:**
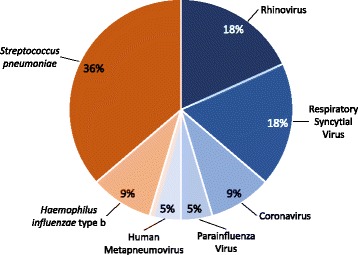
Proportion of respiratory pathogens detected in single infection (*N* = 22) among children <5 years of age hospitalized with severe acute respiratory illness in Niger, 2010–2012

Overall, 138/160 (86 %) of children included in the study tested positive for at least one pathogen (viral or bacterial). At least one respiratory virus was detected in 126/160 (78 %) samples and at least one bacterium was detected in 102/160 (64 %) samples. Among the 138 samples positive for any pathogen, 22 (16 %) were positive for a single pathogen. Of these 8 (36 %) were positive for *S. pneumoniae*, 4 (18 %) for RSV and 4 (18 %) for RV, while HMPV, CV, PIV and Hib each accounted individually for <10 % of the single organisms detected (Fig. 1). Among the 116 children in whom ≥2 organisms were detected, both viral and bacterial pathogens were detected in 90 samples (78 %).

Among the 160 samples tested, RSV (*n* = 56; 35 %) was the most frequently detected virus, followed by RV (*n* = 47; 29 %) and PIV types 1–4 (*n* = 39; 24 %) (Table 2). CV, HMPV, HBV, EV and AV were detected individually in <15 % of the specimens. No PV was detected in our study. Of the 20 samples that tested positive for CV, 13 (65 %) were type OC43, 9 (45 %) were type 229E, 6 (30 %) were type NL63 and 4 (20 %) were type HKU1. Of the 39 samples that tested positive for PIV, 9 (23 %) were type 1, 3 (8 %) were type 2, 31 (79 %) were type 3 and 21 (53 %) were type 4. Even though we selected influenza A and B negative samples, we detected 2 INFA (1.2 %) and 4 INFB positive (2.5 %) samples with the Fast Track platform.

**Table 2:** Detection of respiratory viruses among children <5 years of age hospitalized with severe acute respiratory illness in Niger, 2010–2012

Characteristics	Detection rate *n* (%)								
	RV	CV	PIV	HMPV	BV	RSV	EV	AV	Any virus
Total (*N* = 160)	47 (29.4)	20 (12.5)	39 (24.4)	21 (13.1)	21 (13.1)	56 (35.0)	7 (4.3)	10 (6.2)	126 (78.7)
Age group	*p* = 0.878	*p* = 0.718	*p* = 0.024	*p* = 0.048	*p* = 0.302	*p* <0.001	*p* = 0.700	*p* = 0.987	*p* = 0.006
<1 y (N = 90)	26 (28.9)	12 (13.3)	28 (31.1)	16 (17.8)	14 (15.6)	44 (48.9)	3 (3.3)	6 (6.7)	78 (86.7)
1–4 y (*N* = 70)	21 (30.0)	8 (11.4)	11 (15.7)	5 (7.1)	7 (10.0)	12 (17.4)	4 (5.7)	4 (5.7)	48 (68.6)
Season	*p* <0.001	*p* = 0.178	*p* = 0.014	*p* = 0.049	*p* = 0.003	*p* = 0.001	*p* = 0.558	*p* = 0.581	*p* <0.001
Hot (*N* = 64)	16 (25.0)	12(18.8)	24(37.5)	14 (21.9)	10 (15.6)	34 (53.1)	2 (3.1)	6 (9.4)	56 (87.5)
Cold (*N* = 6)	0 (0.0)	1 (16.7)	0 (0.0)	0 (0.0)	0 (0.0)	0 (0.0)	0 (0.0)	0 (0.0)	1 (16.7)
Rainy (*N* = 48)	26 (54.2)	3 (6.2)	7 (14.6)	5 (10.4)	11(22.9)	13 (27.1)	4 (8.3)	3 (6.2)	42 (87.5)
Dry (*N* = 42)	5 (11.9)	4 (9.5)	8 (19.1)	2 (4.7)	0 (0.0)	9 (21.4)	1 (2.4)	1 (2.4)	27 (64.3)

Abbreviations: *RV* Rhinovirus, *CV* Coronavirus (229, 63, 43, HKU), *PIV* Parainfluenza virus (types 1–4), *HMPV* Human metapneumovirus, *RSV* Respiratory Syncytial Virus, *EV* Enterovirus, *AV* Adenovirus

Co-detections with different subtypes were detected in 6 (30 %) and 20 (51 %) of the CV and PIV positive cases, respectively. RSV, PIV and HMPV were detected more frequently in infants <1 year of age compared to children 1–4 years of age (Table 2). RSV, PIV and HMPV were detected more frequently during the hot season (October to mid-December); while RV and HBV were detected more frequently during the rainy season (June to September). The other viruses were detected with similar frequencies across seasons (Table 2).

Among the 160 samples tested, *S. pneumoniae* (*n* = 90; 56 %) was the most frequently detected bacteria, followed by Hib (*n* = 20; 12 %), *S. aureus* (*n* = 18; 11 %) and *C. pneumoniae* (*n* = 4; 2.5 %) (Table 3). *M. pneumoniae* was not detected in our study.

**Table 3:** Detection of respiratory bacteria among children <5 years of age hospitalized with severe acute respiratory illness in Niger, 2010–2012

Characteristics	Detection rate *n* (%)				
	*C. pneumoniae*	*S. aureus*	*H. influenzae* type b	*S. pneumoniae*	Any bacteria
Total (*N* = 160)	4 (2.5)	18 (11.3)	20 (12.5)	90 (56.3)	102 (63.7)
Age group	*p* = 0.632	*p* = 0.659	*p* = 0.117	*p* = 0.602	*p* = 0.431
<1 y (*N* = 90)	3 (3.3)	11 (12.2)	8 (8.9)	49 (54.4)	55 (61.1)
1-4 y (*N* = 70)	1 (1.4)	7 (10.0)	12 (17.1)	41 (58.6)	47 (67.1)
Season	*p* = 0.467	*p* = 0.976	*p* = 0.245	*p* = 0.579	*p* = 0.748
Hot (*N* = 64)	3 (4.7)	8 (12.5)	7 (10.4)	38 (59.4)	42 (65.6)
Cold (*N* = 6)	0 (0.0)	0 (0.0)	0 (0.0)	3 (50.0)	3 (50.0)
Rainy (*N* = 48)	0 (0.0)	5 (10.4)	4 (8.3)	29 (60.4)	32 (66.7)
Dry (*N* = 42)	1 (2.4)	5 (11.9)	9 (21.4)	20 (47.6)	25 (59.5)

## Discussion

We report the detection rate of selected viral and bacterial pathogens among children <5 years of age hospitalized with SARI in Niger. We detected respiratory viruses in 78 % of our study sample. The high detection rate of viruses found in our study is consistent with results from similar studies conducted in Burkina Faso (73 %) [23], Kenya (68 %) [24] and Brazil (85 %) [12]. However, lower rates of viral detection were reported from other studies from countries such as Ghana (26 %) [25], China (56 %) [26] and Egypt (60 %) [4]. These differences can be attributed to different climatic conditions, enrollment criteria, case definitions and testing platforms.

In our study, RSV was the predominant virus detected and was most commonly found in children <1 year of age. RSV has been reported to be an important pathogen in children and especially in young infants in several studies [12, 25–29]. In addition, RSV detection has been reported to be strongly associated with illness from studies comparing symptomatic cases to controls [30]. Rhinovirus was the second most commonly detected virus (29 %) with similar rates among infants <1 year of age and children aged 1–4 years, which has been reported in previous studies [31, 32]. However, other studies reported RV as the most prevalent virus among children <5 years of age [16, 23, 28, 33]. In addition RV has been commonly detected among asymptomatic persons in several studies indicating that RV can act as both pathogen and by-stander, consequently hindering the ability to infer an association between detection and illness [34–36]. Among the PIV and CV detected in this study, PIV type 3 and CV type OC43 were the most common virus types, which has been reported in other studies [12, 16].

We also found a high detection rate of bacterial pathogens. *S. pneumoniae* (56 %) and Hib (12 %) were the most common bacteria detected in nasopharyngeal specimens. Elevated colonization rates of these bacteria have been reported in children, but only a proportion of colonizations result in invasive disease [9, 11, 37]. The high detection rate of *S. pneumoniae* in our study is likely due to the fact that *S. pneumoniae* is a commensal of the nasopharynx [38]. It has been shown that the prevalence of *S. pneumoniae* carriage in healthy children <5 years of age ranges from 20 % to 93 % in low income countries [11]. The detection of *S. pneumoniae* from sterile sites like blood or cerebrospinal fluid, reflecting invasive pneumococcal disease, has been shown to be lower (5–9 %) [8, 39]. Nonetheless, *S. pneumoniae* has been reported to be responsible for 12 % of meningitis cases in Niger based on cerebrospinal fluid testing; 26 different serotypes were detected among cases of meningitis prior to the introduction of the pneumococcal conjugate vaccine in 2014 [40]. Hib and *S. aureus,* the 2^nd^ and 3^rd^ most prevalent bacterial pathogens in our study, have also been shown to be commensal organisms with high nasopharyngeal carriage rates especially in young children [11]. The substantial Hib nasopharyngeal colonization density found in this study should be investigated further as Hib vaccine has been available in the Niger expanded immunization program since 2008.

Nasopharyngeal specimens may be used to aid in the diagnosis of certain bacterial respiratory pathogens that do not tend to colonize the nasopharynx, such as *M. pneumoniae* and *C. pneumoniae* [38, 41]. *C. pneumoniae* was detected at low rates (2.5 %), and *M. pneumoniae* was not detected in our study. Using serological methods, prevalence rates as high as 30 % have been reported for *C. pneumoniae* [9]; in contrast, other studies report significant detection of *M. pneumoniae* (>10 %) and low detection of *C. pneumoniae* (<1 %) [12, 26, 42].

In our study we found an elevated prevalence (78 %) of viral–bacterial co-detections, which has been reported in other studies [12, 42]. It has been shown that viral infections may predispose to bacterial super-infection by favoring bacterial attachment sites on nasopharyngeal epithelial cells and through increased mucous production that promotes bacterial growth [38, 42].

Our study has limitations that warrant discussion. First, the small sample size of our study hindered our ability to accurately assess the seasonality of the pathogens included in our study. Nonetheless, our results suggest that RSV, PIV and HMPV are more commonly detected during the hot season (October to December), while RV and HBV are detected more frequently during the rainy season (June to September). No difference in the detection rate of the other viruses and bacteria was noted across seasons in our study. The small sample size of our study also hindered our ability to detect patterns of co-detection and the association between specific viral and bacterial co-detections. Second, we did not keep formal records of the proportion of patients consenting to participate in the SARI surveillance. However, a review of the performance of the surveillance system implemented through hospital record review at sentinel sites revealed that only a few patients that met the study case definition were missed by the surveillance program. Third, the lack of controls in our study limited our ability to assess the association of pathogen detection with disease. While most of the viral and bacterial pathogens identified in this study have been described by previous studies as causative agents of ARI, the assignation of causality remains challenging [39, 43]. Fourth, influenza-positive samples were excluded from our study. Co-detection of other viral and bacterial pathogens with influenza is expected and this may have resulted in an underestimation of the prevalence of the pathogens included in this study, especially during the cold season when the majority of influenza-positive cases were detected. Last, we did not systematically collect information on progression of illness (including in-hospital outcome) or risk factors for severe disease, which hindered our ability to evaluate pathogen contribution to the more severe spectrum of illness or to identify groups at high risk for severe disease.

## Conclusion

This study reports the detection rate of viral and bacterial pathogens among children <5 years of age hospitalized with SARI in Niger. The high prevalence of certain viral and bacterial pathogens highlights the need for expanded surveillance in Niger so as to inform policies and interventions. Given the high RSV detection rate observed in this study and the reported association of RSV detection with illness [30], RSV should be included in routine surveillance programs in Niger. Other selected pathogens could be considered for routine surveillance in the country following further assessment to determine association with illness. In addition, information on progression of illness, including in-hospital outcome and risk factors for severe disease should be collected routinely through the existing surveillance system.

## Abbreviations

SARI: Severe acute respiratory illness
FTD: Fast Track Diagnostic
rRT-PCR: real-time reverse transcription polymerase chain reaction
ARIs: Acute respiratory infections
CERMES: Centre de Recherche Médicale et Sanitaire
INF: Influenza
PIV: Parainfluenza virus
CV: Coronavirus
HMPV: Human metapneumovirus
RSV: Respiratory syncytial virus
PV: Parechovirus
EV: Enterovirus
AV: Adenovirus
HBV: Bocavirus
RV: Rhinovirus
*M. pneumoniae*: *Mycoplasma pneumoniae*
*C. pneumoniae*: *Chlamydophila pneumoniae*
*S. pneumoniae*: *Streptococcus pneumoniae*
Hib: *Haemophilus influenzae type b*
*S. aureus*: *Staphylococcus aureus*
